# Improving Pelvic Floor Muscle Training Adherence Among Pregnant Women: Validation Study

**DOI:** 10.2196/30989

**Published:** 2022-02-03

**Authors:** Aida Jaffar, Sherina Mohd-Sidik, Chai Nien Foo, Novia Admodisastro, Sobihatun Nur Abdul Salam, Noor Diana Ismail

**Affiliations:** 1 Department of Psychiatry Faculty of Medicine and Health Sciences Universiti Putra Malaysia Selangor Malaysia; 2 Primary Care Unit Faculty of Medicine and Defence Health Universiti Pertahanan Nasional Malaysia Wilayah Persekutuan Malaysia; 3 Department of Population Medicine Universiti Tunku Abdul Rahman Selangor Malaysia; 4 Software Engineering & Information System Department Faculty of Computer Science & Information Technology Universiti Putra Malaysia Selangor Malaysia; 5 School of Multimedia Technology and Communication College of Arts and Sciences Universiti Utara Malaysia Kedah Malaysia; 6 Klinik Kesihatan Bt 9 Cheras Ministry of Health Selangor Malaysia

**Keywords:** User-centered design, mHealth app, Digital intervention, mHealth Development and Evaluation Framework, Usability, Acceptability, Pelvic Floor Muscle Training, Urinary incontinence, Pregnancy

## Abstract

**Background:**

Mobile health apps, for example, the Tät, have been shown to be potentially effective in improving pelvic floor muscle training (PFMT) among women, but they have not yet been studied among pregnant women. Adherence to daily PFMT will improve pelvic floor muscle strength leading to urinary incontinence (UI) improvement during the pregnancy.

**Objective:**

This study aims to document the validation process in developing the Kegel Exercise Pregnancy Training app, which was designed to improve the PFMT adherence among pregnant women.

**Methods:**

We utilized an intervention mapping approach incorporated within the mobile health development and evaluation framework. The framework involved the following steps: (1) conceptualization, (2) formative research, (3) pretesting, (4) pilot testing, (5) randomized controlled trial, and (6) qualitative research. The user-centered design-11 checklist was used to evaluate the user-centeredness properties of the app.

**Results:**

A cross-sectional study was conducted to better understand PFMT and UI among 440 pregnant women. The study reported a UI prevalence of 40.9% (180/440), with less than half having good PFMT practice despite their good knowledge. Five focus group discussions were conducted to understand the app design preferred by pregnant women. They agreed a more straightforward design should be used for better app usability. From these findings, a prototype was designed and developed accordingly, and the process conformed to the user-centered design–11 (UCD-11) checklist. A PFMT app was developed based on the mHealth development and evaluation framework model, emphasizing higher user involvement in the application design and development. The application was expected to improve its usability, acceptability, and ease of use.

**Conclusions:**

The Kegel Exercise Pregnancy Training app was validated using a thorough design and development process to ensure its effectiveness in evaluating the usability of the final prototype in our future randomized control trial study.

## Introduction

### Background

Urinary incontinence (UI) is defined as an involuntary urinary leakage [1] and has been affecting up to 57.7% of women in low-income countries [2]. Pregnancy affects hormonal changes, which may add an additional risk of UI among adult women [3]. Additionally, other factors, such as damage to the periurethral, paraurethral, and pubo-urethral connective tissues, may occur during pregnancy, labor and delivery, or with obesity, which may impact the urethra and bladder neck position at rest, leading to UI [4]. Another recent meta-analysis, which included studies from 1998 to 2018, stated that about half of pregnant women (41.0%) experienced UI, and it negatively affects their quality of life (QoL) [5].

Women with UI had an unpredictable demand to use the bathroom and became more aware of the bathroom location when shopping and traveling. They felt fear and easily frustrated when they were unable to get to the bathroom on time [6]. During the nighttime, they visited the bathroom a few times, and their sleep was affected significantly. Interrupted sleep led to problems in occupational functioning, as well as psychological functioning, as it worsened depression [7]. Pregnant women may experience a multidimensional negative impact on QoL, such as social-emotional relationships, physical activities, employment issues, limitation to travel, sleep disruption, and obstacles in performing their prayers [8]. Pregnant women who experience UI during pregnancy reported having 5 times the risk of having UI during their postpartum period [9]. Therefore, there is an urgent need to screen and treat them as early as possible by performing pelvic floor muscle training (PFMT).

PFMT or Kegel exercise is an essential exercise among pregnant women. Strengthening their pelvic floor muscles is recommended as it is minimally invasive and does not involve any complications [1,10]. The advantages of the exercise are to shorten the duration of the second stage of labor, reduce severe perineal lacerations [11], and shorten the painful experience of the postpartum period. Pregnant women should be aware of the benefit and be able to perform the correct techniques of the exercise.

Regretfully, not all pregnant women are aware of PFMT despite UI affecting their daily activities because PFMT uptake may be constrained by the antenatal service provision or challenges in accessing services at a primary care clinic [12]. Pregnant women experience challenges related to PFMT education as it involves an individualized approach, for example, the specific language, different levels of health literacy, and cultural variations [13]. In some cultures, the anatomical involvement may be sensitive to certain women [14], leading to necessary adjustments in disseminating the correct information of PFMT according to those cultures.

Additionally, there may be limited attention toward pelvic floor health during pregnancy provided by health care providers (HCPs) as it may be unclear to whom these professional responsibilities belong [13]. PFMT is actually under the responsibility of a physiotherapist; however, pregnant women are under antenatal care follow-up, which is conducted mainly in primary care clinics where there is limited access to physiotherapists [13]. Moreover, the lack of standardized guidelines, inadequate information, and a lack of continuity of care may result in organizational variation in antenatal care services, which worsens the accessibility and acceptability of PFMT services [13].

Regarding individual factors causing training barriers, only one-tenth of pregnant women seek help due to the misperception that UI will resolve by itself [15], assuming that it is “normal” to have UI, and having a misconception that pelvic training will lead to miscarriage [16]. Hence, women face difficulties in achieving the necessary knowledge and skills, resulting in poor attitude and adherence towards pelvic training [13]. Adherence to daily training is one of the most important prognostic factors for PFMT effectiveness in both the short term and longer term [17]. A new method to disseminate PFMT education is necessary to manage both (individual and HCPs) barriers.

### Mobile Health App

Mobile health (mHealth) is defined as “a medical and public health practice supported by mobile devices, such as mobile phones, patient monitoring devices, personal digital assistants, and other wireless devices” [18]. The National Institute for Health and Care Excellence Guideline has categorized the mHealth apps into three-tier functional classifications:

1. Tier A: An app that provides health and social care services without measurable patient outcomes.
2. Tier B: An app that provides health and lifestyle information, health monitoring, or patient-health care professional communication.
3. Tier C: An app with interventions [19].

With regards to the tier C classification, the mHealth app intervention consists of six items which are (1) addressing preventative behavior change, such as smoking; (2) addressing self-management specific behaviors using behavior change techniques; (3) guiding the treatment; (4) providing active monitoring, for example, tracking patients’ location; (5) providing diagnosis, care, or calculated treatment; and (6) providing or guiding the diagnoses [19].

What makes mHealth a powerful tool for behavior modification are its strengths, which include its ease of access and user-friendliness, resulting in its widespread adoption worldwide [20,21]. mHealth can be used by an individual anywhere, at any time, and patients can communicate with health care providers or even a chatbot on specific issues related to the app they are using [22,23]. The strength of a well-designed app is the ability to be well-accepted by the users, change their attitudes, and reduce the acceptability barriers in receiving health care services.

However, qualitative reviews on midwives reported unfavorable findings which did not fully support the apps for several reasons [24]. Midwives were concerned about the accuracy of the apps [25] and their negative impacts on the patient-professional relationship, such as shifting the patient’s trust from trusted to untrusted sources [24]. Hence, there is a need to design a validated pregnancy mHealth app that has undergone the necessary steps based on a framework to improve its effectiveness.

### User-Centered Design

An mHealth app needs to be carefully designed to ensure its impact on the users and its effectiveness. Despite mHealth becoming popular, there is still limited evidence of its effectiveness [26], which is most probably due to the unmet need of incorporating users in the design process [27]. mHealth apps designed *for* the users but not *with* the users will lead to high rates of technology rejection [28,29]. Hence, mHealth apps should be designed to fulfill the user’s requirement using a user-centered design (UCD) framework.

Designing and developing an mHealth app using the user-centered element by involving the users in all stages has its robustness and is a gold standard approach in accomplishing mHealth apps that are useful, easy to use, and satisfying to the users [30]. For example, an mHealth app on the diet and oral health for parents of preschool children, which was developed based on their needs, included information on how to prevent oral disease in their children and has been scored with good usability [31]. UCD requires an iterative design process to understand and internalize the users’ needs, goals, strengths, limitations, contexts, and intuitive processes [32]. Additionally, the iterative process includes observing the users’ interaction with the app during the development process [32]. After understanding the importance and iterative UCD element, the app is then developed and incorporated within the software development life cycle (SDLC), which has been reported as the most liked technique to develop a high-quality software product [33].

The mHealth development and evaluation framework was developed using the iteration SDLC version, and it includes six stages: (1) conceptualization, (2) formative research, (3) pretesting, (4) pilot testing, (5) randomized controlled trial, and (6) qualitative research for further refinement before moving to a more scaled-up intervention [34].

Conceptualization involved experts’ decisions regarding the theoretical basis, reviewing the evidence, and planning the development process via several brainstorming sessions. The research team includes a persuasion element to improve the user’s engagement with the app.

Persuasive systems may be defined as “computerized software or information systems designed to reinforce, change, or shape attitudes or behaviors or both without using coercion or deception” [35]. the persuasive system design (PSD) categorized the 28 strategies into four main categories: primary task support, dialogue support, credibility support, and social support [35]. Among the most PSD used was the primary task support using the self-monitoring tracking [36] in physical activity apps.

Formative research, which is the next stage, involved focus group discussions (FGDs) with pregnant women to determine how they used the app and which design they preferred to use on their mobile phones. The pretesting stage, which stressed the importance of the message context (PFMT adherence), was set and strengthened the collection of responses from pregnant women. The reason for the differences was that not all participants would be able to grasp every message, and key messages (PFMT) could be repeated in different contexts to reach more pregnant women.

A pilot study stage was conducted to obtain further feedback from pregnant women after they had used it over several weeks. The next stage is the randomized control trial stage aimed at obtaining rigorous evidence and, finally, a qualitative study to explore the use of the app in depth.

This study aims to document the validation process of a newly designed mHealth app called the Kegel Exercise Pregnancy Training (KEPT) app running on the Android platform. The KEPT app is intended to deliver training sessions, send reminders, and chart PFMT and UI symptoms. The expectation of the app is to deliver the correct method of PFMT efficiently and conveniently according to the pregnant women’s time and place, without the need to be in the clinic or consult with physiotherapists or doctors. The KEPT app is expected to fill the information gap between clinical visits and has undergone its usability evaluation by the experts [37].

## Methods

### Overview

We incorporated an intervention mapping (IM) approach with a UCD SDLC framework called the mHealth development and evaluation framework. This study focused on conceptualizing the app and using the UCD-11 checklist to evaluate the user-centeredness properties of the app.

### Intervention Mapping

IM is a framework that was designed to plan an effective intervention from the needs assessment up to its evaluation. It comprises stepwise decision-making in developing, implementing, and evaluating interventions using community-based research methods [38,39]. According to Fernandez et al, the participation from the community is to ensure that the intervention matches priority population needs and intervention contexts [39]. The characteristics of this approach involve three aspects that are applied during the intervention planning process: (1) participatory planning, (2) the comprehensive theory used, and (3) an ecological and systems approach for understanding health problems and intervening to address them [40].

Intervention development, according to IM, comprises six steps: (1) demonstrate a comprehensive understanding of the health problem; (2) outline the behavioral and environmental outcomes; (3) identify theory-based and evidence-based behavior change methods that affect the determinants and translate these into practical applications that fit the intervention context; (4) combine the intervention components into a coherent program that uses delivery channels that fit the context; (5) develop implementation strategies to facilitate adoption, implementation, and maintenance of the program; and (6) plan both process and outcome evaluations to assess program implementation, and efficacy or effectiveness [39].

**Figure 1:**
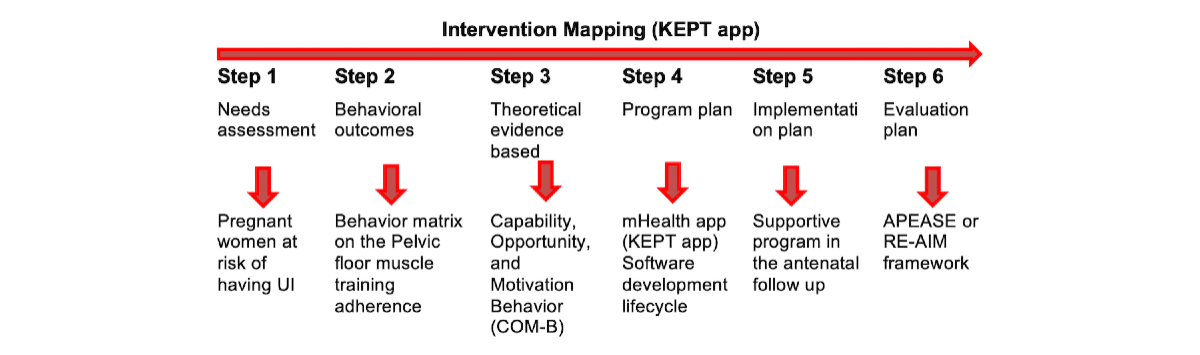
Intervention mapping framework of the KEPT app development. APEASE: affordability, practicability, effectiveness and cost-effectiveness, acceptability, side-effects and safety, equity; KEPT: Kegel Exercise Pregnancy Training; RE-AIM: reach, effectiveness, adoption, implementation, maintenance; UI: urinary incontinence.

Accordingly, the intervention mapping of this mHealth app involved the stepwise approach from the needs assessment followed with other steps as illustrated in Figure 1.

The behavior matrix of this intervention can be divided into knowledge (14 questions), attitude (8 questions), and practices (4 questions) with regards to pelvic floor muscle exercise (PFME). The outcomes of the intention are self-efficacy (17 questions) [41] and adherence (6 questions) [42]. A few examples are listed in Textbox 1. Additional information about the questionnaire has been presented in the results section and published elsewhere [43].

**Figure 2:**
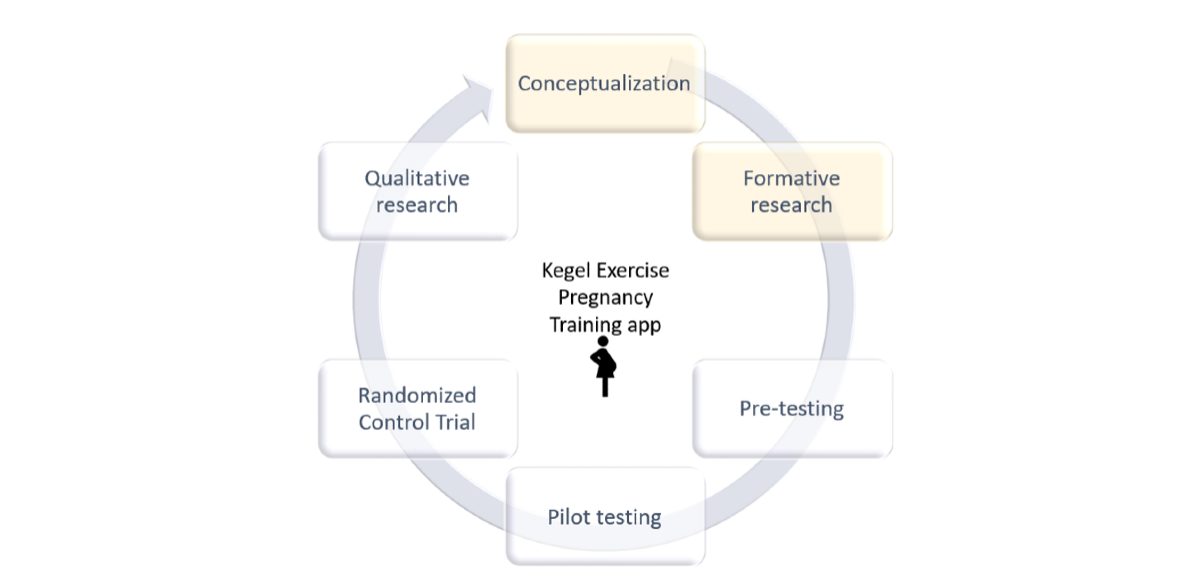
KEPT app development and evaluation framework. KEPT: Kegel Exercise Pregnancy Training.

This study involved documenting the project identification stage followed by the user experience design (Figure 2).

**Behavior matrix of the KEPT app intervention to assess the determinants of pregnant women’s adherence towards pelvic floor muscle training. KEPT: Kegel Exercise Pregnancy Training; PFME: pelvic floor exercise; UI: urinary incontinence.**

**Knowledge (K):**

- Pelvic floor muscles involvement in pelvic exercise.
- Benefits of pelvic exercise.
- Methods in performing the pelvic exercise.

**Attitude (A):**

- I should practice PFME to prevent or treat UI.
- I should practice PFME to prevent uterine prolapsed.
- I feel that PFME is boring.

**Practice (P):**

- I had performed PFME when I was not pregnant.
- I have spent time performing PFME.
- I have tried to search for information regarding PFME.

**Outcomes:**

- Self-Efficacy (SE; how confident you can):
  Perform pelvic exercises on your own.
  Remember to perform exercises every day.
  Perform the exercises at least three times a week.
- Adherence (AD):
  I do my exercises as often as recommended.
  I forget to do my exercises.
  I do fewer exercises than recommended by my health care professional.

In the project identification phase (conceptualization), we determined pregnant women’s (users) understanding of pelvic exercise and the severity of this problem (UI) affecting their QoL. From this foundation, we produced a low-fidelity design, and we conducted FGDs to find out the users’ experience using the design.

### Conceptualization

The team assessed the latest evidence regarding the users' UI and pelvic floor training, finalizing the relevant theoretical behavioral change to be used and planning the development process according to the IM.

### Cross-Sectional Study

A detailed understanding of at-risk pregnant women was established by conducting a cross-sectional study at a primary care clinic [43]. Within this study, we also determined the prevalence and severity of UI and its impact on participants’ QoL at a primary care clinic (user’s study setting) [43,44]. The findings from this study provided input for the content of their educational videos and short notes on PFMT [45,46], which were captured as frequently asked questions (FAQ).

### Behavioral Change Theory

The team brainstormed and decided to identify theory-based and evidence-based behavior change methods concerning PFMT and UI. The interventions were found to be effective when they were developed based on behavioral changed theories, for example, the social cognitive theory and the health belief model [47]. Another overarching framework of behavior used to identify appropriate targets for enhancing adherence in clinical practice is the capability, opportunity, and motivation-behavior (COM-B) model [48,49].

The COM-B model states that motivation is a crucial source of strength to perform a certain behavior, with the assistance from the capability (physical and psychological) and opportunity (physical and social) to engage in the behavior [50], and the strength of motivation to engage in the behavior must be greater than for any other competing behavior.

### Formative Research

A focus group discussion (FGD) study was conducted to understand which design was preferred by the end users. A low-fidelity prototype was given to 5 groups of end users as they chose their preferred design, including their reasoning.

### Ethics Approval

This study was conducted according to the guidelines of the Declaration of Helsinki. The study approvals were obtained in August 2019 from the Ethics Committee for Research Involving Human Subjects, Universiti Putra Malaysia (JKEUPM-2019-368) and the Medical Research and Ethics Committee (MREC), Ministry of Health Malaysia (NMRR-19-412-45606).

## Results

### Conceptualization (Needs Assessment)

#### Cross-Sectional Study

A cross-sectional study was conducted among 440 pregnant women in a semi-urban primary care clinic with a response rate of 72.1% (440/610). The validated study instruments used consisted of socio-demography, knowledge, attitude, and practice on PFME and the International Consultation on Incontinence Questionnaire-Urinary Incontinence Short Form to determine UI among the respondents. The study reported that good knowledge, attitude, and practice scores towards pelvic exercise among pregnant women were 58.0% (255/440), 46.6% (205/440), and 45.2% (199/440), respectively, with further details published elsewhere [43]. The result indicated that pregnant women were not exercising despite having good knowledge (Table 1).

**Table 1:** Socio-demographic data among pregnant women (N=440).

Socio-demographic		Value
Age in years, mean (SD)^a^		29.84 (4.69)
**Ethnicity, n (%)**		
	Malay	356(80.9)
	Chinese	41 (9.3)
	Indian	29 (6.6)
	Others	14 (3.2)
**Financial status, n (%)^b^**		
	Less than RM3000	128 (34.5)
	RM3000-RM6274	176 (47.4)
	RM6275-RM13147	59 (15.9)
	RM13148 and above	8 (2.2)
**BMI, n (%)**		
	Underweight	43 (9.8)
	Normal	143 (32.5)
	Overweight	144 (32.7)
	Obese	110 (25.0)
**History of Cesarean Section, n (%)**		
	No	181 (69.9)
	Yes	78 (30.1)
**Previous history UI^c^, n (%)**		
	No	354 (80.5)
	Yes	85 (19.3)
**Pregnancy category, n (%)**		
	Primigravida	170 (38.6)
	Multigravida	230 (52.3)
	Grand multigravida	40 (9.1)
**Trimester, n (%)**		
	First trimester	53 (12.0)
	Second trimester	152 (34.5)
	Third trimester	235 (53.4)
**Category of UI, n (%)**		
	No UI	260 (59.1)
	Slight UI	95 (21.6)
	Moderate UI	80 (18.2)
	Severe UI	5 (1.1)
**Knowledge on PFME^d^, n (%)**		
	Poor	185 (42.0)
	Good	255 (58.0)
**Attitude towards PFME, n (%)**		
	Poor	235 (53.4)
	Good	205 (46.6)
**Practice on PFME, n (%)**		
	Poor	241 (54.8)
	Good	199 (45.2)

^a^The median age is 30.0 years (27-33).

^b^A currency exchange rate of 1MYR = US $0.24 is applicable. The median income is RM4000 (2000-6000).

^c^UI: urinary incontinence.

^d^PFME: pelvic floor muscle exercise.

Pregnant women were unaware that the pelvic floor muscles were involved in controlling the anus and vagina. Some of them (184/440, 41.8%) were unaware of the correct duration and frequency of the exercise (158/440, 35.9%; Table 2). Hence, pregnant women were unaware of the anatomy of the pelvic floor muscles and the correct techniques of the pelvic exercise.

Only a quarter of them (111/440, 25.2%) strongly agreed that they should be taught the exercise and less than a fifth of them (83/440, 18.9%) agreed to put any effort into doing the exercise and practicing it. Pregnant women’s attitude toward PFME in this study was less favorable (Table 3).

Very few of them (15/440, 3.4%) were performing the exercise whether or not they were pregnant, and only a few of them (12/440, 12.7%) have been practicing the exercise regularly (Table 4).

Based on this cross-sectional study, there was a need to have the PFMT intervention in delivering the correct pelvic exercise knowledge and self-efficacy improvement among pregnant women in order to improve their pelvic floor muscle strength.

**Table 2:** Pregnant women’s responses about their knowledge of pelvic floor muscle exercises.

Knowledge on PFME^a^	Correct, n (%)
PFME muscles are situated in the pubic region	228 (51.8)
PFME involves muscles in the anal region	196 (44.5)
Vagina muscles are not involved in PFME	49 (11.1)
PFM^b^ are important in controlling bladder function	296 (67.3)
PFM is not involved in controlling the anus	80 (18.2)
PFM is not involved in tightening the vagina	102 (23.2)
PFME can tighten buttocks muscles	193 (43.9)
PFME can prevent UI^c^ during laughing/sneezing/weight bearing	292 (66.4)
PFME can prevent/treat uterine prolapse	244 (55.5)
PFME can be done at any time	315 (71.6)
PFME can be done while performing daily activities	248 (56.4)
Muscles involved should be contracted for 8 seconds	184 (41.8)
PFM should be contracted 8-10 times per exercise	170 (38.6)
PFME should be done at least 3x a day (morning, afternoon, and night)	158 (35.9)

^a^PFME: pelvic floor muscle exercise.

^b^PFM: pelvic floor muscle.

^c^UI: urinary incontinence.

**Table 3:** Pregnant women’s attitude towards pelvic floor muscle exercise.

Attitude on PFME^a^	Strongly agree, n (%)
PFME should be done by all women	45 (10.2)
I should practice PFME to prevent/treat UI^b^	86 (19.5)
I should practice PFME to prevent uterine prolapse	72 (16.4)
I feel that PFME is boring	3 (0.7)
PFME should be taught to all antenatal mothers at antenatal clinics	111 (25.2)
I support those who want to perform PFME	113 (25.7)
I view that PFME can increase sexual satisfaction	73 (16.6)
I will put in the effort to search for info about PFME	83 (18.9)

^a^PFME: pelvic floor exercise.

^b^UI: urinary incontinence.

**Table 4:** Pregnant women’s practice behavior towards pelvic floor muscle exercises.

PFME^a^ practices	Always, n (%)
I have performed PFME when not pregnant	15 (3.4)
I have spent time performing PFME	12 (12.7)
I have discussed PFME with friends	7 (1.6)
I have tried to search for info about PFME	12 (2.7)

^a^PFME: pelvic floor muscle exercise.

### Conceptualization (Theoretical Framework)

The theoretical framework of choice was the COM-B model with a combination of the PSD. The PSD is used with the COM-B is to reinforce the behavior voluntarily and to shape the attitude towards PFMT.

The COM-B model that was built into this app was expected to motivate pregnant women to perform PFMT regularly. Capability was divided into two subcategories: (1) physical capability, whereby pregnant women were fit to contract the affected muscles to perform PFMT, and (2) psychological capability signifying pregnant women understood the correct method of performing PFMT.

Opportunity also had two categories: (1) physical opportunity, such as the KEPT-app itself, and (2) social opportunity in which pregnant women were able to understand further and perform PFMT at their own time. Meanwhile, motivation has relationship with cognitive ability, which boosted women’s confidence to perform PFMT. There were two types of motivation: (1) reflective motivation in which pregnant women incorporated their thought processes to arrange PFMT to be done three times daily, and (2) automatic motivation in which the pregnant women adopted PFMT as part of their routine.

Additionally, the COM-B was integrated with the persuasiveness of the app (Table 5). The KEPT app should be tailored (primary task support) based on the intensity of the exercise, and users have the opportunity to self-monitor (primary task support). The app should be able to send reminders (dialogue support) to remind the user to perform the exercise at a certain time. The expertise and authority (system-credibility support) involved in developing the app are available in the video to convince the app user. Finally, an app should be designed and developed from credible and trustworthy sources to bolster users’ confidence and trust.

**Table 5:** KEPT^a^ app COM-B^b^ model with persuasive system design.

COM-B model and features of the mHealth^c^ app				Persuasive system design
**Capability**				
	**Psychological**			
		Educational video by a registered physiotherapist with an example patient	System credibility-expertise and authority	
	**Physical**			
		Training timer according to the user’s confidence and capability.	Primary support-tailoring.	
**Opportunity**				
	**Physical**			
		The KEPT app was produced by our local University	System credibility-trustworthiness	
	**Social**			
		Frequent Asked Question (FAQs) to provide further information	Primary support- Tailoring	
**Motivation**				
	**Reflective**			
		Improve the understanding of the risks of pelvic floor muscle weakness by watching the video.	System credibility-expertise and authority	
		Calendar charting of the UI^d^ symptoms	Primary task-self-monitoring	
	**Automatic**			
		Daily reminder to perform PFMT^e^ as their routine behavior.	Dialogue support-reminder	

^a^KEPT: Kegel Exercise Pregnancy Training.

^b^COM-B: capability, opportunity, motivation behavior-model.

^c^mHealth: mobile health.

^d^UI: urinary incontinence.

^e^PFMT: pelvic floor muscle training.

### Formative Research (Focus Group Discussion)

FGDs were conducted among 24 pregnant women at two primary care clinics to understand the desirability of the app design. The participants were invited via purposive sampling while waiting for their modified oral glucose tolerance test. A total of 5 sessions were conducted as listed in Table 6.

**Figure 3:**
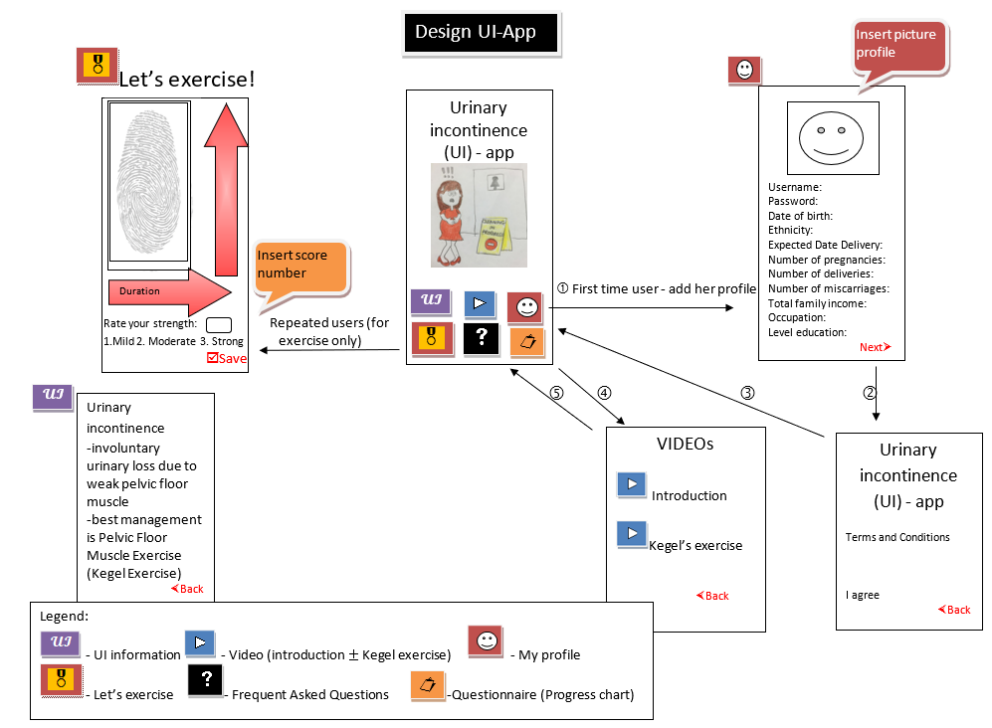
Low-fidelity design of the KEPT app (six user interfaces). KEPT: Kegel Exercise Pregnancy Training.

**Figure 4:**
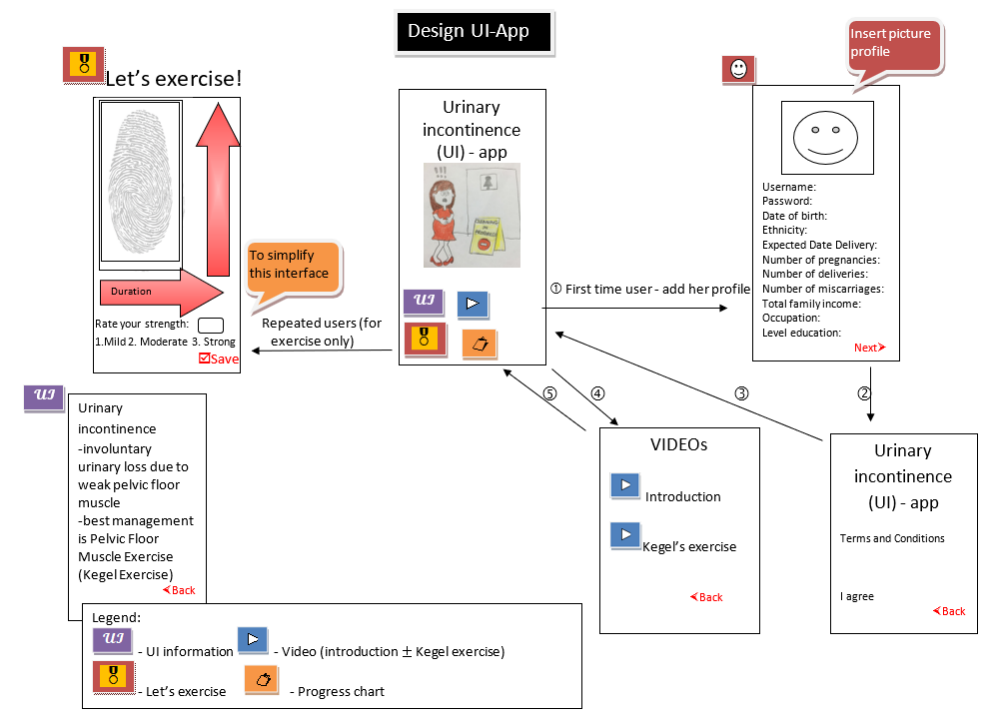
Low-fidelity design of the KEPT app (four user interfaces). KEPT: Kegel Exercise Pregnancy Training.

These interviews were conducted from September to November 2019. The discussion was conducted in a deductive manner to understand the most preferred user interfaces. The questions aimed to make the app as simple as possible since the participants will be using the app 3 times every day. The low-fidelity app designs were provided, and participants could select either Figure 3 (with 6 user interfaces) or Figure 4 (with 4 user interfaces).

Study participants were being asked about their experiences adhering to the exercise:

I have an experience with the PFMT. After I gave birth, I was selected to be followed up by a physiotherapist. The physiotherapist instructed me to perform the PFMT. He then inserted a camera and showed me the muscles contracted. I just performed the exercise. I just know how to perform it, and I do as the physiotherapist instructed to achieve 100 times a day. I did not count it as I do it regularly everyday all the time.

Participant #18

The statement suggested that pregnant women with or without urinary symptoms were motivated to perform and adhere to the exercise even after delivery. A correct understanding of PFMT importance assisted in compliance with daily exercise.

Following this, they were asked to share their opinion regarding adding PFMT notes into the KEPT app. The majority of study participants (19/24, 79.1%) preferred the apps without notes due to the time factor. However, one participant disagreed and mentioned:

…but it is better to have both notes and video. Sometimes, I want to know more about the exercise…

Participant #20

The response suggested mixed opinions on whether or not to include additional notes on the exercise.

**Figure 5:**
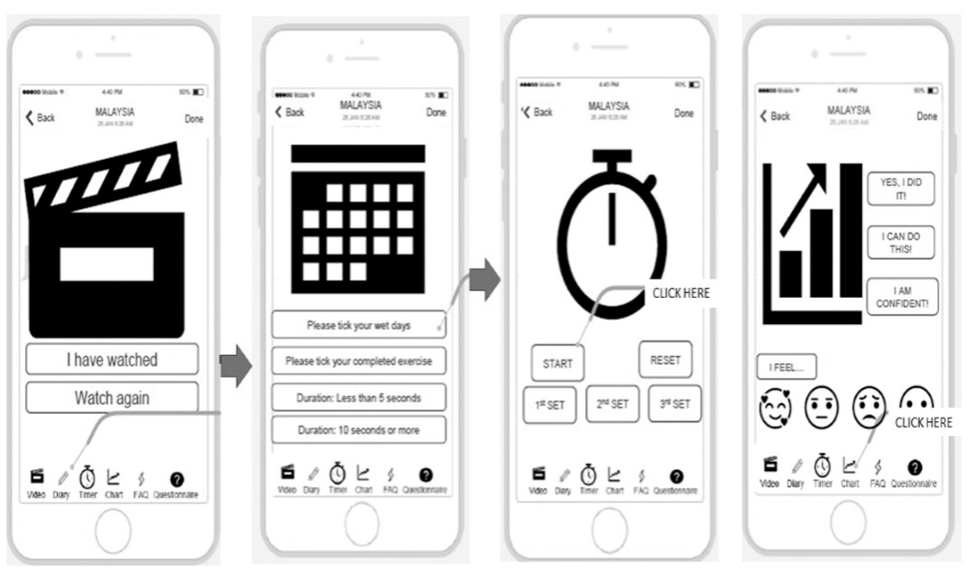
High-fidelity prototype design. KEPT: Kegel Exercise Pregnancy Training.

Finally, regarding the design selection of the app, all the study participants from the FGD preferred design 2 (a more straightforward and minimalist concept). A high-fidelity prototype design was developed based on all the above findings as illustrated in Figure 5.

**Table 6:** Study participants' characteristics and opinions.

ID	Age	Ethnicity	Occupation	UI^a^	PFMT^b^	Timer	FAQ^c^
01	29	Malay	Ex-document manager	Yes	Yes	Yes	No
02	25	Malay	Pharmacist	Yes	No	Yes	No
03	24	Malay	Housewife	No	No	Yes	No
04	21	Malay	Housewife	No	No	Yes	No
05	25	Malay	Graph designer	No	No	Yes	No
06	29	Malay	Housewife	No	No	Yes	No
07	31	Chinese	Engineer	No	No	Yes	No
08	31	Malay	Clerk	No	No	Yes	No
09	31	Malay	Clerk	No	No	Yes	No
10	28	Malay	Clerk	No	No	Yes	No
11	25	Malay	Housewife	No	No	Yes	No
12	23	Indian	Housewife	Yes	No	Yes	No
13	31	Malay	Admin	Yes	No	Yes	No
14	24	Malay	Ex-Assistant Pharmacist	Yes	No	Yes	No
15	27	Malay	Housewife	Yes	No	Yes	No
16	27	Malay	Admin	No	No	Yes	Yes
17	30	Malay	Housewife	No	No	Yes	Yes
18	33	Malay	Staff Nurse	No	Yes	Yes	Yes
19	27	Malay	Housewife	No	No	Yes	Yes
20	21	Malay	Housewife	No	No	Yes	Yes
21	24	Malay	Make-up artist	No	No	Yes	No
22	22	Malay	Housewife	No	No	Yes	No
23	19	Malay	Salesperson	No	No	Yes	No
24	25	Malay	Housewife	No	No	Yes	No

^a^UI: urinary incontinence.

^b^PFMT: pelvic floor muscle exercise.

^c^FAQ: frequently asked questions.

### Prototype Development

**Figure 6:**
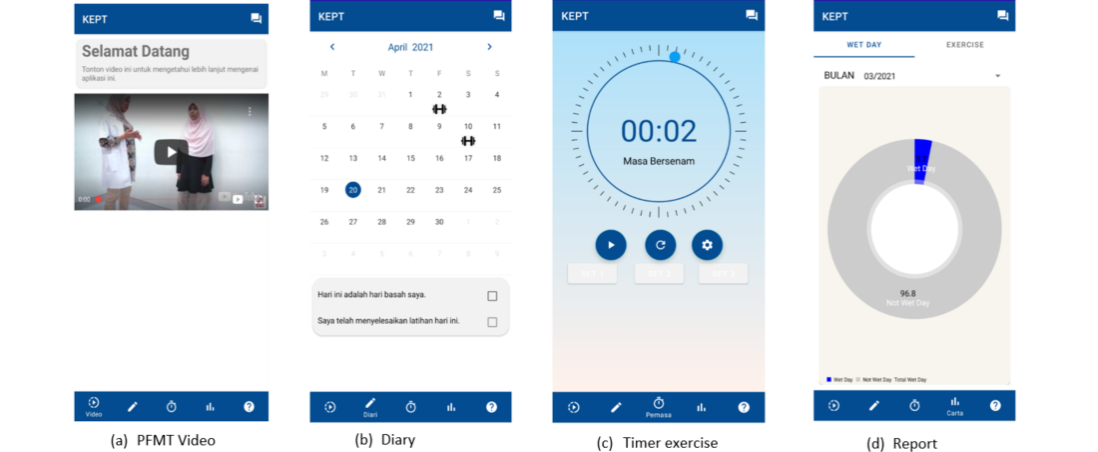
Prototype KEPT app version 1.0. KEPT: Kegel Exercise Pregnancy Training.

The KEPT app prototype was proposed and discussed with the developer. The developer has designed the prototype with a blue color background [37] as illustrated in Figure 6. It consisted of both languages: Malay and English, whereby common English words were used. A minimum number of words were used to ensure the minimalist concept as target users were busy taking care of their families and/or working. There were 2 timer sounds: a high-pitch sound for exercise and a low-pitch sound during 6 seconds of rest. The reminder will be delivered if the PFMT is not completed at 6 pm on the same day.

The KEPT app interface did not require participants to log out due to the need to train three times daily. The UCD-11 checklist has been used as a guide for developing the KEPT app according to its user-centeredness properties as listed in Table 7.

**Table 7:** UCD-11^a^ Items in the KEPT^b^ app

UCD-11 item	KEPT app
Were potential end users (eg, patients, caregivers, family and friends, and surrogates) involved in any steps to help understand users (eg, who they are, in what context might they use the tool) and their needs?	Pregnant women with urinary incontinence are involved in the needs assessment. They will use the app as a supportive tool for self-empowerment to improve their pelvic floor muscle strength.
Were potential end users involved in any steps of designing, developing, and/or refining a prototype	A cross-sectional study to understand the needs assessment [43].
Were potential end users involved in any steps intended to evaluate prototypes or a final version of the tool?	Study protocol for a randomized control trial has been published for this evaluation [51].
Were potential end users asking their opinions of the tool in any way?	Users’ usability testing study has been completed and is currently under the manuscript writing process.
Were potential end users observed using the tool in any way?	Users’ usability evaluation (think aloud method) study has been completed and is currently under the manuscript writing process.
Did the development process have 3 or more iterative cycles?	1. First iterative was the focus group discussion. 2. Second iterative cycle was with the user’s usability study. 3. The third iterative cycle is currently being conducted in a pilot feasibility study [52].
Were changes between iterative cycles explicitly reported in any way?	The users’ usability study is undergoing its manuscript writing process.
Were health professionals asked their opinion of the tool at any point?	The researcher team includes a family medicine specialist, public health specialist, physiotherapist, and community health specialist involved during the development of the app.
Were health professionals consulted before the first prototype was developed?	A family medicine specialist, public health specialist, and physiotherapist were consulted before the first prototype was developed.
Were health professionals consulted between initial and final prototypes?	The research team includes a family medicine specialist, public health specialist, physiotherapist, and community health specialist
Was an expert panel involved?	The research team and the software developer were involved in the development of the app.

^a^UCD-11: user-centeredness design-11.

^b^KEPT: Kegel Exercise Pregnancy Training.

## Discussion

The KEPT app was designed and developed from an IM approach integrated with the mHealth development and evaluation framework. The COM-B model combined with PSD may help to improve the target audience’s “trust” towards the app. Prior to this study, there were few PFMT apps available on the iOS and Android platforms. However, most of the apps had minimum specific strategies for enhancing adherence [53].

The cross-sectional study showed a significant association between UI and knowledge of PFMT (*P*=.01) and attitude towards PFMT (*P*=.01), with poor PFMT practice despite participants’ knowledge was good [43]. This signified that not only are the training techniques but knowledge and attitude are crucial in managing UI among pregnant women. This result was supported by the FGDs, where one group highly insisted on having brief notes regarding PFMT via illustration (ie, the anatomy, physiology, and correct techniques).

Additionally, brief notes or a version of FAQs will serve as an information accuracy checklist, which is included in one of the app trustworthiness checklists [54]. Trustworthiness implies the qualities an individual requires to consider the app as trustable and consists of information accuracy, transparency, organizational attributes, societal influences, and external pressure [54]. Being able to deliver trustable and accurate PFMT techniques will improve PFMT self-efficacy among pregnant women, resulting in improved adherence to PFMT.

Pregnant women in this study also stated that they were unaware of the importance of incorporating good design into mHealth apps monitoring their daily activities, for example, recording the baby’s movements. Nevertheless, with all the actionable features, the KEPT app was designed to enable pregnant women to adopt PFMT as their new instigation habit. Perhaps, in the future, the KEPT app may be upgraded by adding an additional antenatal diary log that may consist of a fetal movement chart, dietary nutrition, and physical activities.

COM-B was used to select the correct intervention (ie, training the pelvic floor muscle) based on previous studies [43,44]. The PSD component of the system’s credibility and trustworthiness [55], with the expertise involved in the development, may add to the user’s sense of safety and reliability regarding the KEPT app. Additionally, the KEPT app’s reminder, self-monitoring, and PFMT timer were discussed. These three components may assist pregnant women in signaling the environment, whereby it is a habit trigger to get prepared for PFMT and go into an automatic mode [56]. Once the habit has been established, a person will be inclined to perform the behavior unconsciously or effortlessly with minimum awareness [57].

The KEPT app was the first app developed for a pregnancy-related target audience from the UCD approach, improving its acceptability and engagement [52]. There was an implication of applying the UCD-11 checklist as it is systematic and comprehensive, which will assist future researchers in developing the mHealth app effectively. However, being iterative for at least three times may have added challenges and financial complications to comply with. The prototype repeated needs in terms of to be evaluation, redevelopment, and re-evaluation may demotivate the researchers and software developers to undergo the iteration again. The KEPT app is currently undergoing pilot testing before entering the randomized control trial phase [51].

This study has a few limitations, such as time constraints and movement restriction orders. Although the study was conducted for an appropriate duration, curfew and restriction movement orders impacted the documentation, administration, and development of the software due to the COVID-19 pandemic situation in Malaysia.

### Conclusions

The KEPT app was developed from a UCD-based behavioral change theory and accompanied by the PSD to improve users’ engagement. The integration of the UCD-11 checklist with COM-B and PSD has prevailed to benefit the target user effectively. Therefore, it is crucial that the targeted users evaluate the usability and user acceptance of the final prototype in our next study.

## Abbreviations

COM-B: capability, opportunity, and motivation-behavior
FAQ: frequently asked question
HCP: health care providers
IM: intervention mapping
KEPT: Kegel Exercise Pregnancy Training
mHealth: mobile health
PFME: pelvic floor muscle exercise
PFMT: pelvic floor muscle training
PMF: pelvic floor muscle
PSD: persuasive system design
QoL: quality of life
SLDC: software development life cycle
UCD: user-centered design
UI: urinary incontinence

## References

[1] Abrams P, Andersson K, Apostolidis A, Birder L, Bliss D, Brubaker L, Cardozo L, Castro-Diaz D, O'Connell P, Cottenden A, Cotterill N, de Ridder D, Dmochowski R, Dumoulin C, Fader M, Fry C, Goldman H, Hanno P, Homma Y, Khullar V, Maher C, Milsom I, Newman D, Nijman RJ, Rademakers K, Robinson D, Rosier P, Rovner E, Salvatore S, Takeda M, Wagg A, Wagner T, Wein A, members of the committees (2018). 6th International Consultation on Incontinence. Recommendations of the International Scientific Committee: Evaluation and Treatment of Urinary Incontinence, Pelvic Organ Prolapse and Faecal Incontinence. Neurourol Urodyn.

[2] Mostafaei H, Sadeghi-Bazargani H, Hajebrahimi S, Salehi-Pourmehr H, Ghojazadeh M, Onur R, Al Mousa RT, Oelke M (2020). Prevalence of female urinary incontinence in the developing world: A systematic review and meta-analysis-A Report from the Developing World Committee of the International Continence Society and Iranian Research Center for Evidence Based Medicine. Neurourol Urodyn.

[3] Sangsawang B (2014). Risk factors for the development of stress urinary incontinence during pregnancy in primigravidae: a review of the literature. European Journal of Obstetrics & Gynecology and Reproductive Biology.

[4] Falah-Hassani K, Reeves J, Shiri R, Hickling D, McLean L (2021). The pathophysiology of stress urinary incontinence: a systematic review and meta-analysis. Int Urogynecol J.

[5] Moossdorff-Steinhauser HFA, Berghmans BCM, Spaanderman MEA, Bols EMJ (2021). Prevalence, incidence and bothersomeness of urinary incontinence in pregnancy: a systematic review and meta-analysis. Int Urogynecol J.

[6] Maeda N, Urabe Y, Suzuki Y, Hirado D, Morikawa M, Komiya M, Mizuta R, Naito K, Shirakawa T (2021). Cross-Sectional Study of the Prevalence and Symptoms of Urinary Incontinence among Japanese Older Adults: Associations with Physical Activity, Health-Related Quality of Life, and Well-Being. IJERPH.

[7] Przydacz M, Skalski M, Sobanski J, Chlosta M, Raczynski K, Klasa K, Dudek D, Chlosta P (2021). Association between Lower Urinary Tract Symptoms and Sleep Quality of Patients with Depression. Medicina.

[8] Al Kiyumi MH, Al Belushi ZI, Jaju S, Al Mahrezi AM (2020). Urinary Incontinence Among Omani Women: Prevalence, risk factors and impact on quality of life. Sultan Qaboos Univ Med J.

[9] Wang K, Xu X, Jia G, Jiang H (2020). Risk Factors for Postpartum Stress Urinary Incontinence: a Systematic Review and Meta-analysis. Reprod Sci.

[10] Woodley SJ, Lawrenson P, Boyle R, Cody JD, Mørkved S, Kernohan A, Hay-Smith EJC (2020). Pelvic floor muscle training for preventing and treating urinary and faecal incontinence in antenatal and postnatal women. Cochrane Database Syst Rev.

[11] Sobhgol SS, Smith CA, Dahlen HG (2020). The effect of antenatal pelvic floor muscle exercises on labour and birth outcomes: a systematic review and meta-analysis. Int Urogynecol J.

[12] Moltu C, Stefansen J, Svisdahl M, Veseth M (2012). Negotiating the coresearcher mandate - service users' experiences of doing collaborative research on mental health. Disabil Rehabil.

[13] Salmon VE, Hay-Smith EJC, Jarvie R, Dean S, Terry R, Frawley H, Oborn E, Bayliss SE, Bick D, Davenport C, MacArthur C, Pearson M (2020). Implementing pelvic floor muscle training in women's childbearing years: A critical interpretive synthesis of individual, professional, and service issues. Neurourol Urodyn.

[14] Grant A, Currie S (2020). Qualitative exploration of the acceptability of a postnatal pelvic floor muscle training intervention to prevent urinary incontinence. BMC Womens Health.

[15] Moossdorff-Steinhauser HFA, Berghmans BCM, Spaanderman MEA, Bols EMJ (2021). Urinary incontinence during pregnancy: prevalence, experience of bother, beliefs, and help-seeking behavior. Int Urogynecol J.

[16] Bayat M, Eshraghi N, Naeiji Z, Fathi M (2021). Evaluation of Awareness, Adherence, and Barriers of Pelvic Floor Muscle Training in Pregnant Women: A Cross-sectional Study. Female Pelvic Med Reconstr Surg.

[17] Dumoulin C, Hay-Smith J, Frawley H, McClurg D, Alewijnse D, Bo K, Burgio K, Chen S, Chiarelli P, Dean S, Hagen S, Herbert J, Mahfooza A, Mair F, Stark D, Van Kampen M, International Continence Society (2015). 2014 consensus statement on improving pelvic floor muscle training adherence: International Continence Society 2011 State-of-the-Science Seminar. Neurourol Urodyn.

[18] Kay M, Santos J, Takane M (2011). mHealth: New horizons for health through mobile technologies. mHealth: New horizons for health through mobile technologies. World Heal Organ.

[19] National Institute for Health Care Excellence (NICE) Evidence Standards Framework for Digital Health Technologies. National Institute for Health and Care Excellence.

[20] Carroll JK, Moorhead A, Bond R, LeBlanc WG, Petrella RJ, Fiscella K (2017). Who Uses Mobile Phone Health Apps and Does Use Matter? A Secondary Data Analytics Approach. J Med Internet Res.

[21] Baharuddin R, Singh D, Razali R (2013). Usability Dimensions for Mobile Applications-A Review. RJASET.

[22] Overdijkink SB, Velu AV, Rosman AN, van Beukering MD, Kok M, Steegers-Theunissen RP (2018). The Usability and Effectiveness of Mobile Health Technology-Based Lifestyle and Medical Intervention Apps Supporting Health Care During Pregnancy: Systematic Review. JMIR Mhealth Uhealth.

[23] Hauser-Ulrich S, Künzli H, Meier-Peterhans D, Kowatsch T (2020). A Smartphone-Based Health Care Chatbot to Promote Self-Management of Chronic Pain (SELMA): Pilot Randomized Controlled Trial. JMIR Mhealth Uhealth.

[24] Vickery M, van Teijlingen E, Hundley V, Smith G, Way S, Westwood G (2020). Midwives’ views towards women using mHealth and eHealth to self-monitor their pregnancy: A systematic review of the literature. Eur J Midwifery.

[25] Soltani H, Furness P, Arden M, McSeveny K, Garland C, Sustar H, Dearden A (2012). Women's and Midwives' Perspectives on the Design of a Text Messaging Support for Maternal Obesity Services: An Exploratory Study. J Obes.

[26] Marcolino MS, Oliveira JAQ, D'Agostino M, Ribeiro AL, Alkmim MBM, Novillo-Ortiz D (2018). The Impact of mHealth Interventions: Systematic Review of Systematic Reviews. JMIR Mhealth Uhealth.

[27] Schnall R, Rojas M, Bakken S, Brown W, Carballo-Dieguez A, Carry M, Gelaude D, Mosley JP, Travers J (2016). A user-centered model for designing consumer mobile health (mHealth) applications (apps). J Biomed Inform.

[28] Osborne CL, Juengst SB, Smith EE (2020). Identifying user-centered content, design, and features for mobile health apps to support long-term assessment, behavioral intervention, and transitions of care in neurological rehabilitation: An exploratory study. British Journal of Occupational Therapy.

[29] Matthew-Maich N, Harris L, Ploeg J, Markle-Reid M, Valaitis R, Ibrahim S, Gafni A, Isaacs S (2016). Designing, Implementing, and Evaluating Mobile Health Technologies for Managing Chronic Conditions in Older Adults: A Scoping Review. JMIR Mhealth Uhealth.

[30] Cornet V, Toscos T, Bolchini D, Rohani Ghahari R, Ahmed R, Daley C, Mirro MJ, Holden RJ (2020). Untold Stories in User-Centered Design of Mobile Health: Practical Challenges and Strategies Learned From the Design and Evaluation of an App for Older Adults With Heart Failure. JMIR Mhealth Uhealth.

[31] Akmal Muhamat N, Hasan R, Saddki N, Mohd Arshad MR, Ahmad M (2021). Development and usability testing of mobile application on diet and oral health. PLoS One.

[32] Witteman HO, Vaisson G, Provencher T, Chipenda Dansokho S, Colquhoun H, Dugas M, Fagerlin A, Giguere AM, Haslett L, Hoffman A, Ivers NM, Légaré F, Trottier M, Stacey D, Volk RJ, Renaud J (2021). An 11-Item Measure of User- and Human-Centered Design for Personal Health Tools (UCD-11): Development and Validation. J Med Internet Res.

[33] Sen S, Patel M, Sharma A (2021). Software Development Life Cycle Performance Analysis. Mathur R, Gupta CP, Katewa V, Jat DS, Yadav N. editors.

[34] Whittaker R, Merry S, Dorey E, Maddison R (2012). A development and evaluation process for mHealth interventions: examples from New Zealand. J Health Commun.

[35] Oinas-Kukkonen H, Harjumaa M, Oinas-Kukkonen H, Hasle P, Harjumaa M, Segerståhl K, Øhrstrøm P (2008). A Systematic Framework for DesigningEvaluating Persuasive Systems BT - Persuasive Technology. Lecture Notes in Computer Science, vol 5033.

[36] Matthews J, Win KT, Oinas-Kukkonen H, Freeman M (2016). Persuasive Technology in Mobile Applications Promoting Physical Activity: a Systematic Review. J Med Syst.

[37] Jaffar A, Sidik SM, Admodisastro N, Mansor EI, Fong LC (2021). Expert’s Usability Evaluation of the Pelvic Floor Muscle Training mHealth App for Pregnant Women. IJACSA.

[38] Kok G, Schaalma H, Ruiter RAC, van Empelen P, Brug J (2004). Intervention mapping: protocol for applying health psychology theory to prevention programmes. J Health Psychol.

[39] Fernandez ME, Ruiter RAC, Markham CM, Kok G (2019). Intervention Mapping: Theory- and Evidence-Based Health Promotion Program Planning: Perspective and Examples. Front Public Heal. (AUG).

[40] Green L, Marshall W (2005). Kreuter: Health Program Planning: An educational and ecological approach.

[41] Sacomori C, Cardoso FL, Porto IP, Negri NB (2013). The development and psychometric evaluation of a self-efficacy scale for practicing pelvic floor exercises. Brazilian Journal of Physical Therapy;.

[42] Newman-Beinart Naomi A, Norton Sam, Dowling Dominic, Gavriloff Dimitri, Vari Chiara, Weinman John A, Godfrey Emma L (2017). The development and initial psychometric evaluation of a measure assessing adherence to prescribed exercise: the Exercise Adherence Rating Scale (EARS). Physiotherapy.

[43] Jaffar A, Mohd-Sidik S, Nien FC, Fu GQ, Talib NH Urinary incontinence and its association with pelvic floor muscle exercise among pregnant women attending a primary care clinic in Selangor, Malaysia. Rosier PFWM, editor. PLoS One. Public Library of Science; 2020.

[44] Jaffar A, Mohd-Sidik S, Abd Manaf Rosliza, Foo C, Gan Q, Saad H (2021). Quality of life among pregnant women with urinary incontinence: A cross-sectional study in a Malaysian primary care clinic. PLoS One.

[45] Alagirisamy P, Mohd Sidik S (2020). Pelvic Floor Muscle Exercises During and After Pregnancy.

[46] Bo K, Berghmans B, Morkved S, Van Kampen M (2014). Evidence-Based Physical Therapy for the Pelvic Floor-E-Book: Bridging Science and Clinical Practice, 2nd ed.

[47] McClurg D, Frawley H, Hay-Smith J, Dean S, Chen S-Y, Chiarelli P (2015). Scoping review of adherence promotion theories in pelvic floor muscle training - 2011 ics state-of-the-science seminar research paper i of iv. Neurourol Urodyn. John Wiley & Sons, Ltd.

[48] Woodley SJ, Hay-Smith EJC (2021). Narrative review of pelvic floor muscle training for childbearing women-why, when, what, and how. Int Urogynecol J.

[49] Michie S, van Stralen MM, West R (2011). The behaviour change wheel: a new method for characterising and designing behaviour change interventions. Implement Sci.

[50] Garnett C, Crane D, West R, Brown J, Michie S (2015). Identification of Behavior Change Techniques and Engagement Strategies to Design a Smartphone App to Reduce Alcohol Consumption Using a Formal Consensus Method. JMIR Mhealth Uhealth.

[51] Sidik SM, Jaffar A, Foo CN, Muhammad NA, Abdul Manaf R, Ismail SIF, Alagirisamy P, Ahmad Fazlah AF, Suli Z, Goodyear-Smith F (2021). KEPT-app trial: a pragmatic, single-blind, parallel, cluster-randomised effectiveness study of pelvic floor muscle training among incontinent pregnant women: study protocol. BMJ Open.

[52] Jaffar A, Mohd Sidik S, Foo CN, Muhammad NA, Abdul Manaf R, Fadhilah Ismail SI Protocol of a Single-Blind Two-Arm (Waitlist Control) Parallel-Group Randomised Controlled Pilot Feasibility Study for mHealth App among Incontinent Pregnant Women. Int J Environ Res Public Health. 2021.

[53] Latorre GFS, de Fraga R, Seleme MR, Mueller CV, Berghmans B (2019). An ideal e-health system for pelvic floor muscle training adherence: Systematic review. Neurourol Urodyn.

[54] van Haasteren A, Gille F, Fadda M, Vayena E (2019). Development of the mHealth App Trustworthiness checklist. DIGITAL HEALTH.

[55] Asklund Ina, Nyström Emma, Sjöström Malin, Umefjord Göran, Stenlund Hans, Samuelsson Eva (2017). Mobile app for treatment of stress urinary incontinence: A randomized controlled trial. Neurourol Urodyn.

[56] Chen W, Chan T, Wong L, Looi C, Liao C, Cheng H, Wong Sl, Mason J, So H, Murthy S, Gu X, Pi Z (2020). IDC theory: habit and the habit loop. RPTEL.

[57] Hagger M (2019). Habit and physical activity: Theoretical advances, practical implications, and agenda for future research. Psychology of Sport and Exercise.

