# Correction: Improving Pelvic Floor Muscle Training Adherence Among Pregnant Women: Validation Study

**DOI:** 10.2196/38175

**Published:** 2022-04-11

**Authors:** Aida Jaffar, Sherina Mohd-Sidik, Chai Nien Foo, Novia Admodisastro, Sobihatun Nur Abdul Salam, Noor Diana Ismail

**Affiliations:** 1 Department of Psychiatry Faculty of Medicine and Health Sciences Universiti Putra Malaysia Selangor Malaysia; 2 Primary Care Unit Faculty of Medicine and Defence Health Universiti Pertahanan Nasional Malaysia Wilayah Persekutuan Malaysia; 3 Department of Population Medicine Universiti Tunku Abdul Rahman Selangor Malaysia; 4 Software Engineering & Information System Department Faculty of Computer Science & Information Technology Universiti Putra Malaysia Selangor Malaysia; 5 School of Multimedia Technology and Communication College of Arts and Sciences Universiti Utara Malaysia Kedah Malaysia; 6 Klinik Kesihatan Bt 9 Cheras Ministry of Health Selangor Malaysia

In “Improving Pelvic Floor Muscle Training Adherence Among Pregnant Women: Validation Study” (JMIR Hum Factors 2022;9(1):e30989), the following errors were noted.

1. Abstract:

In the originally published paper, the first sentence in the abstract was stated as

Mobile health apps, for example, the Tät, have been shown to be potentially effective in improving pelvic floor muscle training (PFMT) among women, but their effectiveness in pregnant women was limited.

This has been corrected to:

Mobile health apps, for example, the Tät, have been shown to be potentially effective in improving pelvic floor muscle training (PFMT) among women, but they have not yet been studied among pregnant women.

2. Methods, Intervention Mapping:

The originally published paper was missing two references for this statement:

The outcomes of the intention are self-efficacy (17 questions) and adherence (6 questions).

This has been corrected to:

The outcomes of the intention are self-efficacy (17 questions) (41) and adherence (6 questions) (42).

3. Methods, Cross-Sectional Study:

The originally published paper was missing two references for this statement:

The findings from this study provided input for the content of their educational videos and short notes on PFMT, which were captured as frequently asked questions (FAQ).

This has been corrected to:

The findings from this study provided input for the content of their educational videos and short notes on PFMT (45, 46) which were captured as frequently asked questions (FAQ).

4. Results:

The originally published paper stated the following in row 1, column 2 of Table 5:

1.System credibility-expertise and authority.

2. Primary support-Virtual rehearsal principle

This has been corrected to:

System credibility-expertise and authority

5. Results:

The originally published paper stated the following as the title for the first column of Table 5:

COM-B model and behavioral change techniques incorporated in the mHealth app.

This has been corrected to:

COM-B model and features of the mHealth app.

6. Discussion:

The originally published paper was missing one reference for this statement:

The PSD component of the system’s credibility and trustworthiness, with the expertise involved in the development, may add to the user’s sense of safety and reliability regarding the KEPT app.

This has been corrected to:

The PSD component of the system’s credibility and trustworthiness (55), with the expertise involved in the development, may add to the user’s sense of safety and reliability regarding the KEPT app.

6. References:

In the corrected paper, the following citations have been newly added to the Reference List. As these new references have been numbered per the order of their in-text citations, the remaining citations in the reference list have been renumbered accordingly.

41. Sacomori C, Cardoso FL, Porto IP, Negri NB. The development and psychometric evaluation of a self-efficacy scale for practicing pelvic floor exercises. Brazilian Journal of Physical Therapy; 2013. [doi: 10.1590/S1413-35552012005000104]

42. Newman-Beinart NA, Norton S, Dowling D, Gavriloff D, Vari C, Weinman JA, Godfrey EL. The development and initial psychometric evaluation of a measure assessing adherence to prescribed exercise: the Exercise Adherence Rating Scale (EARS). Physiotherapy, 103(2); 2017, 180–185. [doi: 10.1016/j.physio.2016.11.001]

45. Alagirisamy P, Mohd Sidik S. Pelvic Floor Muscle Exercises During and After Pregnancy. Universiti Putra Malaysia Press Serdang 2020.

46. Bo K, Berghmans B, Morkved S, Van Kampen M. Evidence-Based Physical Therapy for the Pelvic Floor-E-Book: Bridging Science and Clinical Practice, 2nd ed. London, UK: Elsevier Health Sciences; 2014. 3.

55. Asklund I, Nyström E, Sjöström M, Umefjord G, Stenlund H, Samuelsson E. Mobile app for treatment of stress urinary incontinence: A randomized controlled trial. Neurourol Urodyn 2017 Jun;36(5):1369-1376. [doi: 10.1002/nau.23116] [Medline: 27611958]

The correction will appear in the online version of the paper on the JMIR Publications website on April 11, 2022, together with the publication of this correction notice. Because this was made after submission to PubMed, PubMed Central, and other full-text repositories, the corrected article has also been resubmitted to those repositories.

